# Small Antisense RNA RblR Positively Regulates RuBisCo in *Synechocystis* sp. PCC 6803

**DOI:** 10.3389/fmicb.2017.00231

**Published:** 2017-02-14

**Authors:** Jinlu Hu, Tianpei Li, Wen Xu, Jiao Zhan, Hui Chen, Chenliu He, Qiang Wang

**Affiliations:** ^1^School of Life Sciences, Northwestern Polytechnical UniversityXi'an, China; ^2^Key Laboratory of Algal Biology, Institute of Hydrobiology, the Chinese Academy of SciencesWuhan, China; ^3^University of the Chinese Academy of SciencesBeijing, China; ^4^Crop Designing Centre, Henan Academy of Agricultural SciencesZhengzhou, China; ^5^State Key Laboratory of Freshwater Ecology and Biotechnology, Institute of Hydrobiology, the Chinese Academy of SciencesWuhan, China

**Keywords:** sRNA, RblR, RuBisCo, *rbcL*, *Synechocystis* sp. PCC 6803

## Abstract

Small regulatory RNAs (sRNAs) function as transcriptional and post-transcriptional regulators of gene expression in organisms from all domains of life. Cyanobacteria are thought to have developed a complex RNA-based regulatory mechanism. In the current study, by genome-wide analysis of differentially expressed small RNAs in *Synechocystis* sp. PCC 6803 under high light conditions, we discovered an asRNA (RblR) that is 113nt in length and completely complementary to its target gene *rbcL*, which encodes the large chain of RuBisCO, the enzyme that catalyzes carbon fixation. Further analysis of the RblR(+)/(−) mutants revealed that RblR acts as a positive regulator of *rbcL* under various stress conditions; Suppressing RblR adversely affects carbon assimilation and thus the yield, and those phenotypes of both the wild type and the overexpressor could be downgraded to the suppressor level by carbonate depletion, indicated a regulatory role of RblR in CO_2_ assimilation. In addition, a real-time expression platform in *Escherichia coli* was setup and which confirmed that RblR promoted the translation of the *rbcL* mRNA into the RbcL protein. The present study is the first report of a regulatory RNA that targets RbcL in *Synechocystis* sp. PCC 6803, and provides strong evidence that RblR regulates photosynthesis by positively modulating *rbcL* expression in *Synechocystis*.

## Introduction

Small regulatory RNAs (sRNAs) are key genetic regulators in organisms from all domains of life. In bacteria, these regulatory RNAs are generally referred to as sRNAs, because they usually range from 50 to 500 nt in length (Gottesman and Storz, 2011). These sRNAs control a variety of processes, including chromosome maintenance (Storz, 2002; Volpe et al., 2002), the stability and translation of mRNAs (Storz et al., 2004), the stability and translocation of proteins (Huttenhofer et al., 2005; Hüttenhofer and Vogel, 2006), stress responses (Romby et al., 2006), metabolic reactions (Park et al., 2010), and pathogenesis (Lee and Groisman, 2010). The most extensively studied sRNAs, often referred to as trans-encoded sRNAs or intergenic region-sRNAs (IGRs), are those that map onto intergenic regions and regulate target RNAs via short, only partially complementary base pairing interactions. In Gram-negative bacteria, the RNA-binding protein Hfq is usually required for the function and/or stability of sRNAs. *Cis*-encoded antisense sRNAs (asRNAs) that are located on the strand of DNA opposite their mRNA targets, exhibit extensive complementarity to their targets. The base pairing of asRNAs with their mRNA counterparts has typically either negative or positive regulatory effects on their mRNA targets (Raghavan et al., 2012; Sakurai et al., 2012). In addition to sRNAs, bacteria contain some regulatory elements within the 5′ leader regions of mRNAs, such as riboswitches, which regulate gene expression by adopting different conformations in response to external and internal factors (Smith et al., 2010; Phok et al., 2011; Ramesh et al., 2011). These elements modulate transcriptional elongation, mRNA stability, and the initiation of translation following exposure to specific stimuli (Coppins et al., 2007).

Regulatory sRNAs in cyanobacteria have been identified by computational prediction and subsequent experimental verification (Axmann et al., 2005; Voss et al., 2009; Ionescu et al., 2010), microarray-based approaches (Steglich et al., 2008; Georg et al., 2009; Gierga et al., 2012), and sRNA sequencing (sRNA-Seq; Mitschke et al., 2011a,b; Waldbauer et al., 2012; Billis et al., 2014; Kopf et al., 2014; Pfreundt et al., 2014; Voigt et al., 2014; Xu et al., 2014). sRNA-Seq, an unbiased method that allows the entire sRNA repertoire in any organism to be investigated, is the most powerful approach for sRNA identification (Liu et al., 2009). This technique can be performed without prior knowledge of sequences or structural conservation, and overcomes many of the technical limitations of previous approaches (i.e., the low expression levels and small size of sRNA, the limited knowledge of predictable transcriptional signals, and the general lack of robust algorithms to predict sRNAs) (for reviews, see Backofen and Hess, 2010), thereby offering a direct, efficient approach for identifying sRNAs in bacteria.

Cyanobacteria constitute a wide variety of photoautotrophic bacteria and are present in almost all environments, including fresh water, oceans, rock surfaces, desert soil, and the polar regions (Schopf, 1993). As cyanobacteria use sunlight as their sole energy source, these organisms are exposed to a particular set of environmental challenges not endured by other bacteria (Kopf and Hess, 2015). All cyanobacteria have developed extensive regulatory systems that involve regulatory proteins and RNA-based elements. In addition, sRNAs are an essential component of regulatory systems, since they, in principle, allow the system to have an individual regulator at a very low cost compared to protein regulators, and they undergo rapid state transitions in regulatory networks, which is supported by various dynamic simulations (Shimoni et al., 2007; Mehta et al., 2008). In cyanobacteria, cis-encoded asRNA transcripts appear to be very dominant in a number of cyanobacteria, e.g., asRNAs summing up to 26% of all genes for the unicellular *Synechocystis* sp. PCC 6803 (Georg et al., 2009; Mitschke et al., 2011a) and to 39% of all genes in the nitrogen-fixing *Anabaena* sp. PCC 7120 (Mitschke et al., 2011b) were reported. The all asRNAs reported so far are cis-encoded chromosomal RNAs in cyanobacteria. Thus, chromosomally encoded asRNAs may have an important function in cyanobacterial regulatory networks. The *Synechocystis* transcriptome includes over 4,000 transcriptional units, close to half of which were thought represent sRNAs, most of which were also considered as asRNAs (Kopf et al., 2014). It is found that at least five sRNAs regulate photosynthetic gene expression in *Synechocystis*, including IsrR, As1-Flv4, PsbA2R, PsbA3R, and PsrR1, and all of these except PsrR1 are asRNAs (Dühring et al., 2006; Eisenhut et al., 2012; Sakurai et al., 2012; Georg et al., 2014). Interestingly, these asRNAs appear to have repressive (IsrR and As1-Flv4) and activating (PsbA2R and PsbA3R) effects on gene expression.

The activity of ribulose-1,5-bisphosphate carboxylase/oxygenase (RuBisCO), the most abundant enzyme in nature, is considered to be the main limiting factor of photosynthesis in C3 plants (Farquhar et al., 1980), C4 plants (Furbank et al., 1996), and green algae (Bassham and Krause, 1969). RuBisCO assimilates inorganic carbon into the biosphere and catalyzes the carboxylation and oxygenation of ribulose-1,5-bisphosphate (RuBP) in photosynthesis and photorespiration, respectively (Luo et al., 2002; Marcus et al., 2011). Given its importance, it is not surprising that RuBisCO is the most abundant protein in leaves, accounting for 50% of the soluble leaf protein in C3 plants and 30% in C4 plants (Feller et al., 2008). In land plants and cyanobacteria, the *rbcL* gene encodes the large chain of RuBisCO (Spreitzer and Salvucci, 2002). Moreover, the binding sites of the enzymatically active substrate (RuBP) are located in RbcL proteins that form homodimers (Berg et al., 2012).

In this study, we identified *Synechocystis* sRNAs using sRNA-Seq technology under normal light (NL) and high light (HL) conditions. This analysis allowed us not only to detect and quantify transcripts of known sRNAs, but also to identify previously unidentified sRNAs. Moreover, we experimentally verified some known and predicted sRNAs to increase our understanding of sRNAs in *Synechocystis*. In addition, we investigated the asRNA RblR under various growth conditions related to photosynthesis. By complementary base pairing, RblR appears to positively regulate the *rbcL* gene, which encodes the large chain of RuBisCO.

## Materials and methods

### Strains and growth conditions

Wild-type *Synechocystis* sp. PCC 6803 was grown at 30°C in BG11 medium under continuous illumination (~30 μE·m^−2^·s^−1^). Different growth and stress conditions were applied to exponentially growing *Synechocystis* cultures (OD_750_ 0.6–0.8) to allow all types of RNA to be expressed. The cultures used for sRNA-Seq were grown under NL conditions (exponential growth phase, OD_750_ 0.8, ~30 μE·m^−2^·s^−1^) or transferred to HL conditions (~300 μE·m^−2^·s^−1^) for 24 h, after which they were harvested by centrifugation at 3,000 g (25°C, 5 min), flash frozen in liquid nitrogen, and stored at −80°C.

The mutant strains were subjected to four different stress treatments. For HL stress, samples were collected 12 and 24 h after a shift in light intensity from 30 to 300 μE·m^−2^·s^−1^. For low light (LL) conditions, the samples were collected at 1, 2, and 3 days after the shift from 30 to 2 μE·m^−2^·s^−1^. For heat treatment (HT), the samples were collected 1, 2, and 3 days after a temperature shift from 30 to 42°C. For depleted carbon (−C) conditions, exponentially growing cultures were transferred to carbon-free BG11 (BG11 w/o NaCO_3_, pH 7.0) for 8 h without aeration after two washes in carbon-free BG11.

### RNA extraction, library construction, and deep sequencing

Total RNA from each independent sample subjected to NL or HL conditions was isolated using TRIzol reagent (Invitrogen, USA), according to the manufacturer's instructions. In each case, the extracted product was digested with DNase I (TAKARA, China) to eliminate genomic DNA, followed by rRNA removal using a Ribo-Zero rRNA Removal Kit (Epicenter, USA). The sRNA libraries were prepared using a TruSeq Small RNA Sample Prep Kit (Illumina, USA), following the manufacturer's instructions. Briefly, 1 mg of total RNA was ligated to adapters at the 3′ and 5′ ends without size fractionation. Adapter-ligated RNA was reverse-transcribed using SuperScript II Reverse Transcriptase (Invitrogen, USA) and then PCR-amplified (98°C for 30 s; 10 cycles of 98°C for 10 s, 65°C for 30 s, 72°C for 30 s; 72°C for 5 min). Transcripts ≤ 200 nt in size were selected in a 6% denaturing polyacrylamide gel. The quality and concentration of each cDNA library were evaluated using an Agilent 2100 Bioanalyzer DNA 1000 Assay (Agilent, USA). The cDNA libraries were sequenced using an Illumina Genome Analyzer IIx (Illumina, USA). The deep sequencing data are available from the NCBI Sequence Read Archive under accession number SRR935472.

### Analysis of sequencing data

A total of 7,951,189 and 8,677,859 raw reads were obtained using Solexa sequencing technology from the two different sRNA-Seq samples. The two cDNA libraries were examined for the presence of 3′ and 5′ adaptors, and the adaptor sequences were trimmed from all screened reads. Sequences shorter than 18 nt were designated “short” and were not assigned to the *Synechocystis* genome. The remaining 6,976,872 and 7,764,659 “clean reads” were mapped onto the *Synechocystis* chromosome and its four megaplasmids using the Burrows-Wheeler Alignment (BWA) tool (Li and Durbin, 2009). A filtering procedure was implemented in PerlScript to extract the BWA output.

Much of the extracted BWA output was similar, sometimes differing by only the addition of several nucleotides at the ends. A process described by Liu et al. was performed to remove this source of variation from the data and to unify similar sequences into putative transcripts (Liu et al., 2009). A total of 6,127,890 and 6,650,647 transcripts were obtained from the raw NL and HL sequences, respectively. Each read of merged transcripts was classified according to the following coordinates: annotated open reading frames (ORFs), transcripts from intergenic regions (IGRs), transcripts antisense to ORFs (ASs), transfer RNA (tRNA), and ribosomal RNA (rRNA). Those transcripts that mapped onto IGR and AS were further analyzed.

### RNA blot analysis

RNA samples (50 μg) were denatured for 10 min at 65°C in loading buffer (TAKARA, China). The treated RNA was separated on 10% urea-polyacrylamide gels for 1.5 h at 120 V after a pre-run for 0.5 h at 200 V and transferred to Hybond-N nylon membranes (Amersham, Germany) by electroblotting for 1 h at 300 mA. Gene-specific oligonucleotides were labeled with [γ-^32^P]ATP (PerkinElmer, USA) by the exchange reaction of T4 polynucleotide kinase (NEB, USA) using 10 U of enzyme, 10 pmol oligonucleotide, and 40 μCi [γ-^32^P]ATP in reaction buffer for 1 h at 37°C. The membranes were UV-crosslinked at 1,200 J and the blots were prehybridized at 45°C for 1 h in Hybridization Solution (#HYB-101, TOYOBO, Japan). Hybridization with specific [γ-^32^P]ATP end-labeled oligonucleotides was then performed overnight at 45°C. The membranes were washed in 0.1% SDS in 5X SSC (3 M NaCl, 0.3 M sodium citrate, pH 7.0) at 45°C, followed by 0.1% SDS in 1X SSC for 30 min per wash. Signals were detected and analyzed using a Cyclone Plus Phosphor Imager (#C431200, PerkinElmer, USA). All DNA oligonucleotides used for RNA blot analysis are listed in Supplementary Table 1.

### 5′- and 3′-rapid amplification of cDNA ends (RACE)

Total RNA was digested with DNase I (TAKARA, China) for 30 min at 37°C. The reactions were stopped by phenol chloroform extraction, followed by ethanol precipitation. Precipitated RNAs were redissolved in DEPC H_2_O and prepared for 5′ and 3′ RACE. For 5′ RACE, reverse transcription was performed at 37°C for 60 min using gene-specific primers and SuperScriptIII RNase (Invitrogen, USA), according to the manufacturer's instructions. The 3′ linker was ligated to the 3′ end of purified cDNA with T4 RNA ligase (Fermentas, China) for 48 h at 22°C. The products were amplified using the 3′ linkerPCRrev primer and gene-specific primers. The cycling conditions were as follows: 95°C for 5 min; 40 cycles of 95°C for 30 s, 55°C for 30 s, 72°C for 30 s; and 72°C for 7 min. The products were separated on 2% agarose gels, and bands of interest were excised, gel-eluted using a GeneJET Gel Extraction Kit (#K0691, Themo Scientific, Canada), and cloned into the pMD18-T vector (#6011, TAKARA, China). Colonies obtained after transformation were screened for the presence of PCR products of the appropriate size by colony PCR, followed by sequencing. The 3′ RACE assays were carried out essentially as described by Argaman et al. (2001) with the following modifications. First, ligation with the 3′ linker was performed as described above for 12 h at 16°C. Then, phenol chloroform-extracted, ethanol-precipitated RNA was reverse-transcribed with 100 pmol of a single 3′ linkerRTrev primer complementary to the 3′ linker. PCR amplification, cloning, and sequence analysis were performed as described above. All enzymatic treatments of RNA were performed in the presence of 10 U of RNase Inhibitor (Fermentas, China). All oligonucleotides and primers used for RACE analysis are listed in Supplementary Table 2.

### qRT-PCR validation

Reverse transcription of total RNA was performed with random primer (6 mer) or a reverse primer specific to the sRNA candidate using a PrimeScript RT Reagent Kit with gDNA Eraser (TAKARA, China), according to the manufacturer's instructions. The resulting cDNA was used as a template for PCR amplification with forward and reverse primers specific to each candidate or its target. PCR was performed with an initial denaturation step of 1 min at 95°C, followed by 40 cycles of 5 s at 95°C, 30 s at 60°C, and 65–95°C melting-curve analysis using iTaq Universal SYBR Green Supermix (#172-5120, BIO-RAD, USA). Control reactions were performed for each run and for included RNA samples not treated with reverse transcriptase or samples lacking template DNA. In all cases, no band was observed in these controls. All primers used for the analysis are listed in Supplementary Table 3. All values represent the means of five biological replicates.

### Mutagenesis

The *slr0168* gene and 600 bp of upstream sequence were amplified from the *Synechocystis* genome and cloned into the pMD18-T vector using slr0168-F/slr0168-R primers. Then, the fragments of the *rnpB* promoter and the *Kanamycin* gene produced in a two-step PCR process using primers 5′ rnpB, 3′ rnpB/kana, 5′ kana/rnpB, and 3′ kana were ligated into the *EcoRI* site of *slr0168* to obtain pAB106. The *rbcL* promoter and RblR were subcloned into the pMD18-T vector using Prbcl-F/Prbcl-R and RblR(+)-F/RblR(+)-R primers, followed by excision by *SacI*/*XbaI* digestion, and insertion into the *EcoRV* site of pAB106 to obtain the plasmid used for overexpression of RblR. The plasmid expressing the anti-RblR fragment was constructed by fusing the complementary RblR sequence and the *oop* terminator from bacteriophage lambda using PCR products amplified with the RblR(−)-F and RblR(−)/oop ter-R primers. The control and RblR(+)/RblR(−) vector harboring the different fragments were transferred to wild-type (WT) cells by homologous recombination (Golden et al., 1987), and transformants were selected on BG11 agar plates containing 20 μg/ml kanamycin. Segregations were evaluated by PCR using the primers 0168-F/0168-R.

To construct a real-time simulation platform in *E. coli*, the RblR, anti-RblR, and *rbcL* fragments were amplified from the *Synechocystis* genome and cloned into pMD18-T using RblR(+)-F/RblR(+)-R, RblR(−)-F/RblR(−)-R/oop ter, and *rbcL*-F/*rbcL*-R primers to obtain pAB116, pAB117, and pAB123, respectively. The sequences of three tandem translation terminators and a ribosome-binding site were imported into the 5′ end of *rbcL*-F for *rbcL* gene expression. The *rbcL* gene was then excised by *SacI*/*SalI* digestion and inserted into the *XbaI* site of pAB116/pAB117 to obtain the plasmid for overexpression/knockdown of RblR, namely pAB124/pAB125. All primers used for this analysis are listed in Supplementary Table 4.

### Protein gel and immunoblot analysis

Protein gel and immunoblot analysis were performed as previously described (Hu et al., 2014). The membranes were probed with rabbit primary anti-RbcL antibody (1:10,000).

### Chl fluorescence analysis

Chl fluorescence was measured with a Dual-PAM-100 Chl fluorescence photosynthesis analyzer (Walz, Germany) using 3 ml culture, grown under NL or -C conditions for 8 h at room temperature in darkness, according to the manufacturer's instructions, at which point the cells were in the exponential growth phase. All cultures were enriched to OD_730_ 1.0 by centrifugation at 2,500 g (25°C, 5 min). Absorbance of whole cells was measured with a UV-1800 PC spectrophotometer (MAPADA, China). All values represent the means of five biological replicates.

## Results

### High-resolution transcriptomes of *Synechocystis* sRNAs using dRNA-Seq

**Figure 1:**
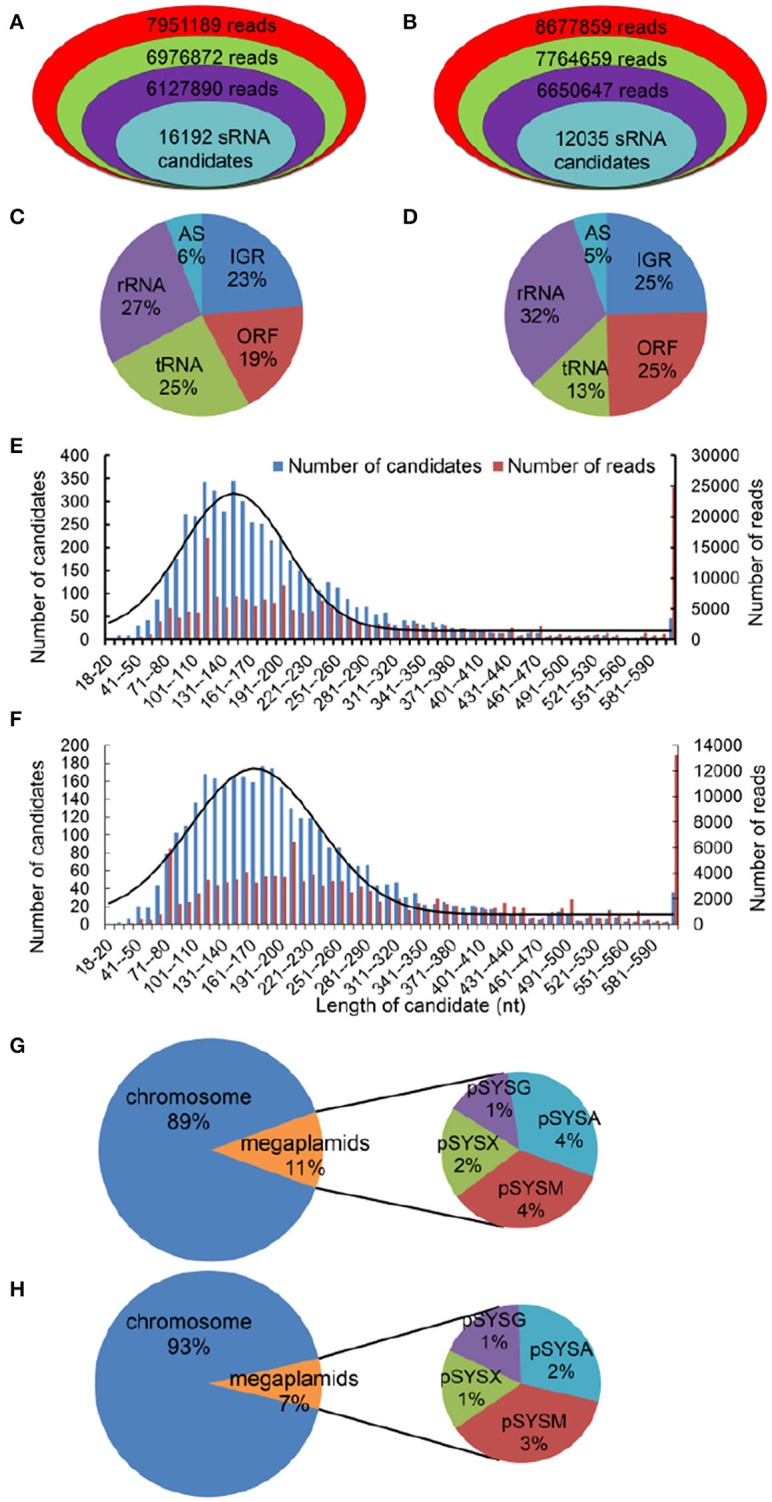
**High-resolution transcriptomes of ***Synechocystis***, as determined by sRNA-seq**. Visual representation of the depth of the sRNA-Seq data reads under NL **(A)** and HL **(B)** conditions. Reads that represent total sequence are in red; reads that are larger than 18 nt are in green; reads that match the *Synechocystis* genome are in purple. All the number of sRNAs (turquoise) after filtering were amongst these candidates. Breakdown of total *Synechocystis* reads based on their genomic origin under NL (**C**, *n* = 6,127,890) and HL (**D**, HL, *n* = 6,650,647) conditions. ORF, annotated open reading frames; AS, transcripts antisense to known genes; IGR, transcripts from intergenic regions. Length distribution of sRNA candidates (≥10 reads) and total reads. The 5,261 and 3,379 sRNA candidates (≥10 reads) under NL **(E)** and HL **(F)** conditions, corresponding to 197,304 and 125,023 total reads, are plotted based on the length of the most abundant sequence observed for each candidate. The fitted gaussian distribution is indicated in black. Location distribution of two sRNA databases, under NL **(G)** and HL **(H)** conditions, in the *Synechocystis* chromosome and its four megaplasmids (pSYSA, pSYSG, pSYSM, and pSYSX).

Using the dRNA-Seq protocol (Sharma et al., 2010), we isolated total RNA from cells cultivated under NL or HL conditions and used this RNA to prepare cDNA libraries enriched for primary transcripts. To focus on sRNAs, we purified the amplicons (≤ 200 nt) used for sequencing using denaturing polyacrylamide gel electrophoresis on 6% polyacrylamide gels. A total of 7,951,189 and 8,677,859 raw reads under NL and HL conditions, respectively, were obtained using Solexa sequencing technology. After discarding low-quality reads with average Phred scores of <20 and sequencing reads shorter than 18 nt, we found that 6,127,890 and 6,650,647 reads matched the *Synechocystis* chromosome and its four megaplasmids (Figures 1A,B). All 6,127,890 and 6,650,647 reads were grouped into putative transcripts by combining sequences with genomic coordinate information (Figures 1C,D). Overall, 52% (27 + 25%) and 45% (32 + 13%) of the total *Synechocystis* reads analyzed were derived from rRNA or tRNA under NL and HL conditions (Figures 1C,D), respectively. The results suggest that the rRNA removal step was reliable for eliminating the majority of these highly abundant transcripts. Between one-fifth and one-quarter of all transcripts corresponded to ORFs (Figures 1C,D). The remaining 29% (23 + 6%) and 30% (25 + 5%) transcripts of the NL and HL databases were mapped to non-annotated regions corresponding to intergenic regions and to transcripts in antisense orientation to known genes (Figures 1C,D).

After filtering, 16,192 and 12,035 sRNA candidates were identified (Figures 1A,B) under NL and HL conditions, respectively. To reduce the rate of the false positives, we further filtered out transcripts with <10 uniquely aligned reads from putative sRNA candidates and obtained 5,261 and 3,380 sRNA candidates, respectively. Notably, 43 of the 89 sRNAs registered in the BSRD (Bacterial Small Regulatory RNA Database; Li et al., 2013) were detected in the current study, indicating the reliability of our method. The lengths of the final set of sRNA candidates (≥10 reads) were mainly concentrated in the 60–310 nt range, and a distribution diagram revealed a normal distribution pattern centered at 146 nt and 167 nt with the standard deviation of 55 nt and 67 nt for NL and HL, respectively (Figures 1E,F), which also implies the reliability of our methods. The number of sRNA candidates and reads of various length differed significantly between the NL and HL databases (Figures 1E,F). In addition, by analyzing the distribution of the two sRNA databases in the *Synechocystis* genome and its four megaplasmids (pSYSA, pSYSG, pSYSM, and pSYSX), we determined that sRNA candidates in megaplasmids represented 11 and 7% of the sRNA databases under NL and HL conditions respectively (Figures 1G,H). These results suggest that sRNA candidates in megaplasmids also participate in the gene expression regulatory network under HL stress conditions.

### Identification of *Synechocystis* sRNA candidates

**Figure 2:**
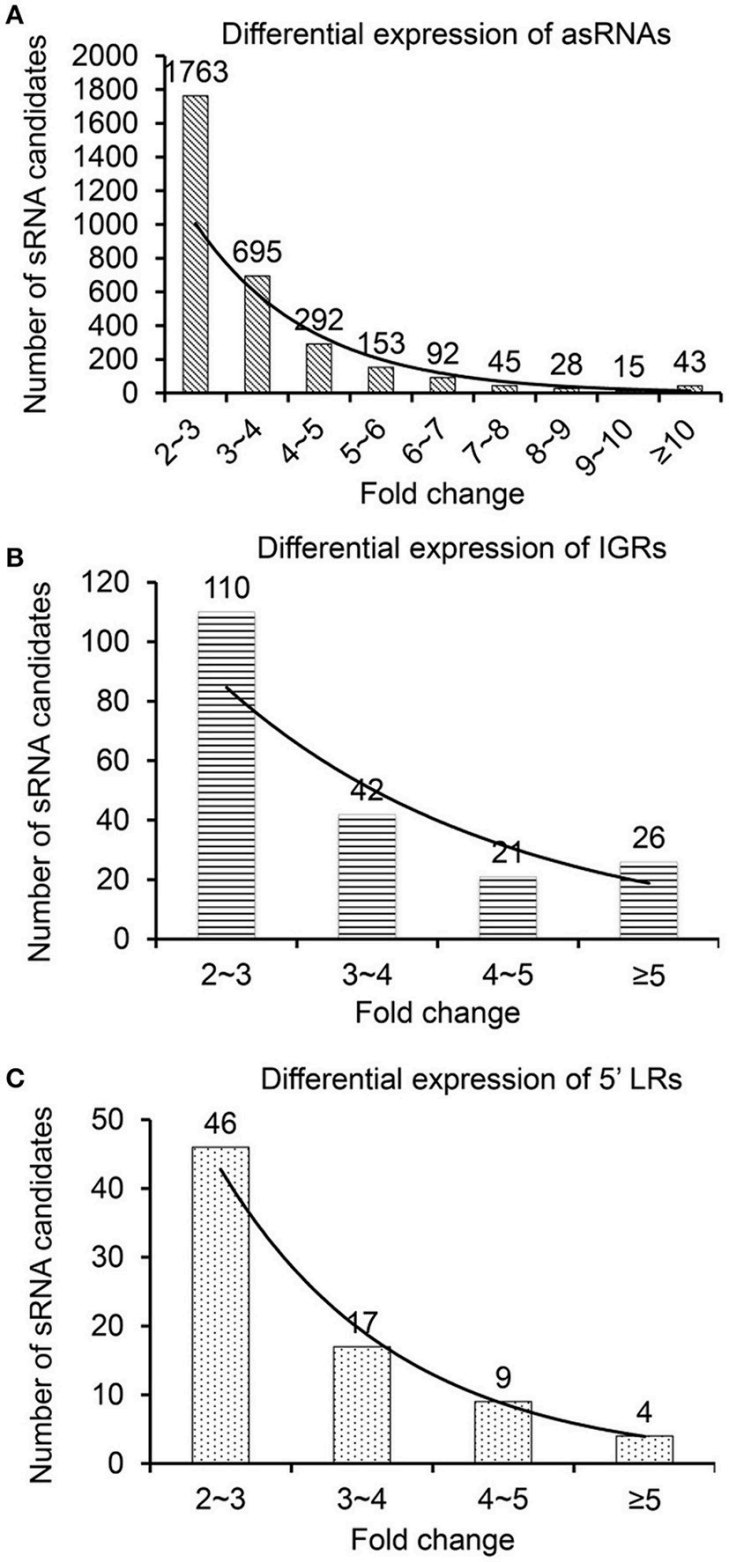
**Fold change of differentially expressed sRNAs under NL and HL conditions**. Up/down-regulated sRNAs of **(A)** asRNAs, **(B)** IGRs, and **(C)** 5′ LRs in the NL and HL libraries.

All sRNA candidates (≥10 reads) were classified into the following three types: IGRs, asRNAs, and 5′ LRs located 300 nt upstream of an annotated gene, according to Xu et al. (2014). In total, we identified 4,664 asRNAs, 433 IGRs, and 164 5′ LRs under NL conditions and 3,009 asRNAs, 284 IGRs, and 87 5′ LRs under HL conditions (Supplementary Figure S1). To observe the expression patterns of sRNAs under NL and HL conditions, we analyzed the two sRNA databases according to the rate of sequence overlap (overlap) and the ratio of differential expression (ratio) for each sRNA considered. Differentially expressed sRNAs in the two sRNA databases had to meet the following three requirements: overlap of ≥10%, ratio of ≥2, and ≥10 reads under NL or HL conditions. Differential expression was mainly described as up/downregulation and fold change in expression of sRNA reads in NL and HL conditions (Supplementary Table 5, Figure 2), and the distribution of the fold change of all categories of the differentially expressed sRNAs, i.e., asRNAs (Figure 2A), IGRs (Figure 2B) and 5′ LRs (Figure 2C) show nicely exponential decay, indicate reliable data and analysis. Using these criteria, we filtered 853 up-regulated sRNAs and 2,548 down-regulated sRNAs in the differential expression libraries. Besides, we identified 1,319 and 670 sRNAs as unique sRNAs under NL and HL databases, respectively (Supplementary Table 5). These results suggest that some sRNAs have interesting expression patterns and that our approach represents an easy way to investigate the influence of HL stress in *Synechocystis*.

**Figure 3:**
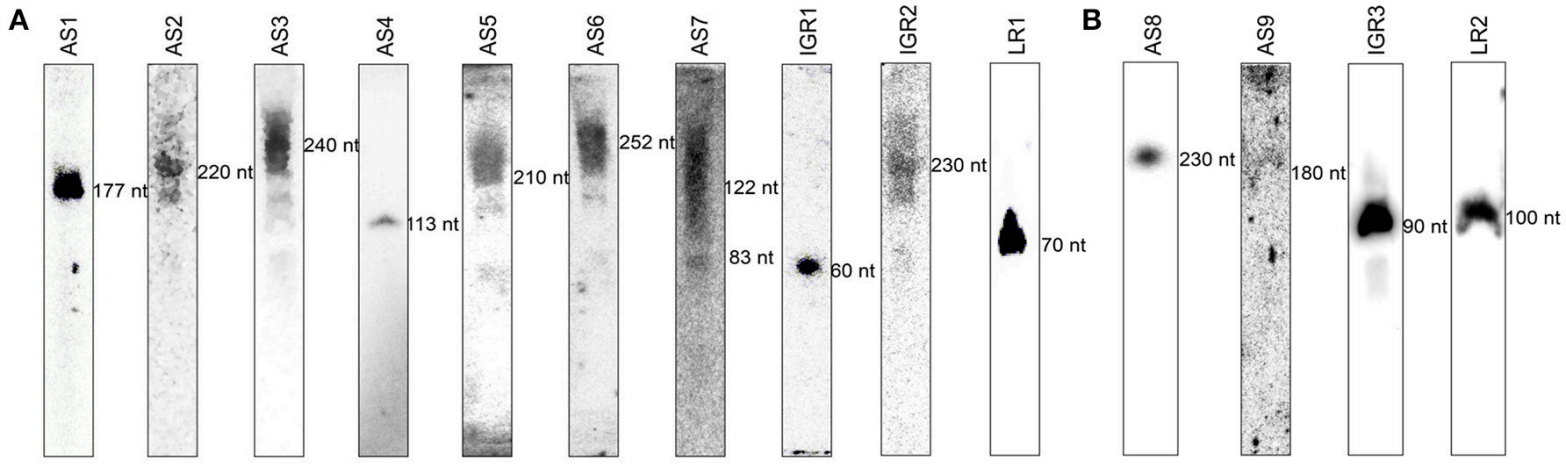
**Detection of novel and established sRNAs by RNA blot hybridization**. Fifty micrograms of total RNA was loaded per lane on a 10% polyacrylamide gel that was electro-blotted on Hybond-N nylon membranes. All sRNAs were detected with an oligonucleotide probe in the differential expression library **(A)** and unique sRNA library under NL conditions **(B)**.

To verify the existence of these candidates *in vivo* and to confirm the reliability of our method, 14 representative sRNA candidates including 3 from different megaplasmids and 11 from the chromosome (Supplementary Table 6) were selected and subjected to further verification by RNA blot hybridization (Figure 3), and among those, the full length of 8 were successfully obtained by RACE (Supplementary Table 6). All previously studied sRNAs in *Synechocystis* (isrR, Yfr2b, and SRP RNA ffs Dühring et al., 2006; Voss et al., 2009) were also identified and verified in our study and designated as AS1, 5′ LR1, and 5′ LR2 (Supplementary Table 6, Figure 3), respectively. The results indicate that sRNA-Seq is a successful technique for identifying sRNAs in *Synechocystis*. Notably, although with exactly the same length, the coordinate of AS1 has a 2 nt shift toward the 5′ end (Supplementary Table 6) as compared with that of isrR (Dühring et al., 2006). For AS7, one transcription start site (TSS) was found at position 1,603,634 on the chromosome, and two 3′ ends were detected at position 1,603,755 and 1,603,716, with fragment lengths of 122 nt and 83 nt, respectively. The abundance of AS7 was extremely low, and the bands produced by RNA blot analysis were weak but present (Figure 3A and Supplementary Table 6).

To confirm the relationship between the asRNAs and their targets, we quantified the expression of eight differentially expressed asRNAs and their corresponding target mRNAs under NL and HL conditions (Supplementary Figure S2). The transcript accumulation of isrR and *isiA* mRNA exhibited a strict inverse relationship (Supplementary Figure S2a). Compared to isrR, seven other asRNAs showed more moderate regulatory effects on target gene expression (Supplementary Figures S2b–h). Five asRNAs had a slightly inverse relationship with their target mRNAs (Supplementary Figures S2b,c,e,g,h), and two asRNAs had positive regulatory effects (Supplementary Figures S2d,f). These results should be interpreted with caution for the following reasons: (1) distinct functions have been identified only for strongly expressed asRNAs, but many sRNAs have relatively low abundance and therefore may simply constitute some type of “transcriptional noise” or may provide some selective advantage; and (2) gene expression is co-regulated by many factors *in vivo* in addition to sRNAs.

### RblR regulates photosynthetic gene expression

**Figure 4:**
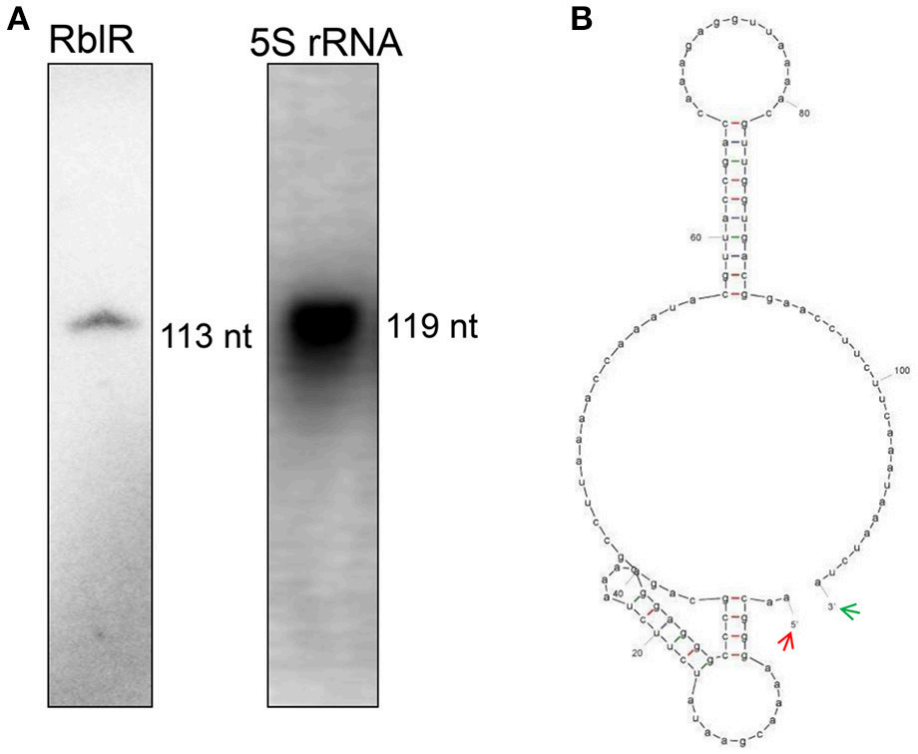
**Identification of the asRNA RblR. (A)** Typical detection pattern of RblR and 5S rRNA by RNA blot analysis. Five micrograms of total RNA was loaded for detection of 5S rRNA. Eighty micrograms of total RNA was applied to the lane labeled RblR. **(B)** RNA secondary structure prediction for RblR. Arrows indicate experimentally detected RNA 5′ (red) and 3′ (green) ends.

We subjected one of the sRNA candidates to functional characterization during photosynthesis. In this analysis, the asRNA AS4 was named RblR, since its sequence is antisense to its target *rbcL* mRNA, which encodes the large subunit of RuBisCO, the enzyme that catalyzes carbon fixation. The length of RblR was validated as being 113 nt by RACE (Supplementary Table 6) and RNA blot analysis (Figures 3A, 4A), and its 5′ end was mapped to nucleotide c2 478,718 in the totally sequenced genome of this organism. Thus, RblR extends from position 299 to position 411 with regard to the coding sequence of *rbcL*. However, unlike *rbcL* mRNA, RblR is present at relatively low levels, according to both sRNA-Seq and RNA blot analysis. RblR can be folded into two extended stem region ending with a terminal loop (Figure 4B). Such loop structures are frequently involved in RNA-RNA interactions and thus may be functionally relevant for its hypothetical trans-acting function. However, it is more likely that RblR has a direct effect on *rbcL* mRNA levels.

To further investigate the relationship between RblR and the corresponding *rbcL* gene, we constructed normal (back ground control), overexpressor and suppressor mutants of RblR, referred to as control, RblR(+) and RblR(−), respectively (Supplementary Figure S3a). The *rnpB* and *rbcL* promoter-driven platform together with a *kanamycin* resistance cassette were inserted into the neutral site *slr0168* to obtain the RblR(+) and RblR(−) strains, while the control strain contains only the *kanamycin* resistance cassette (Supplementary Figures S3a–c).

**Figure 5:**
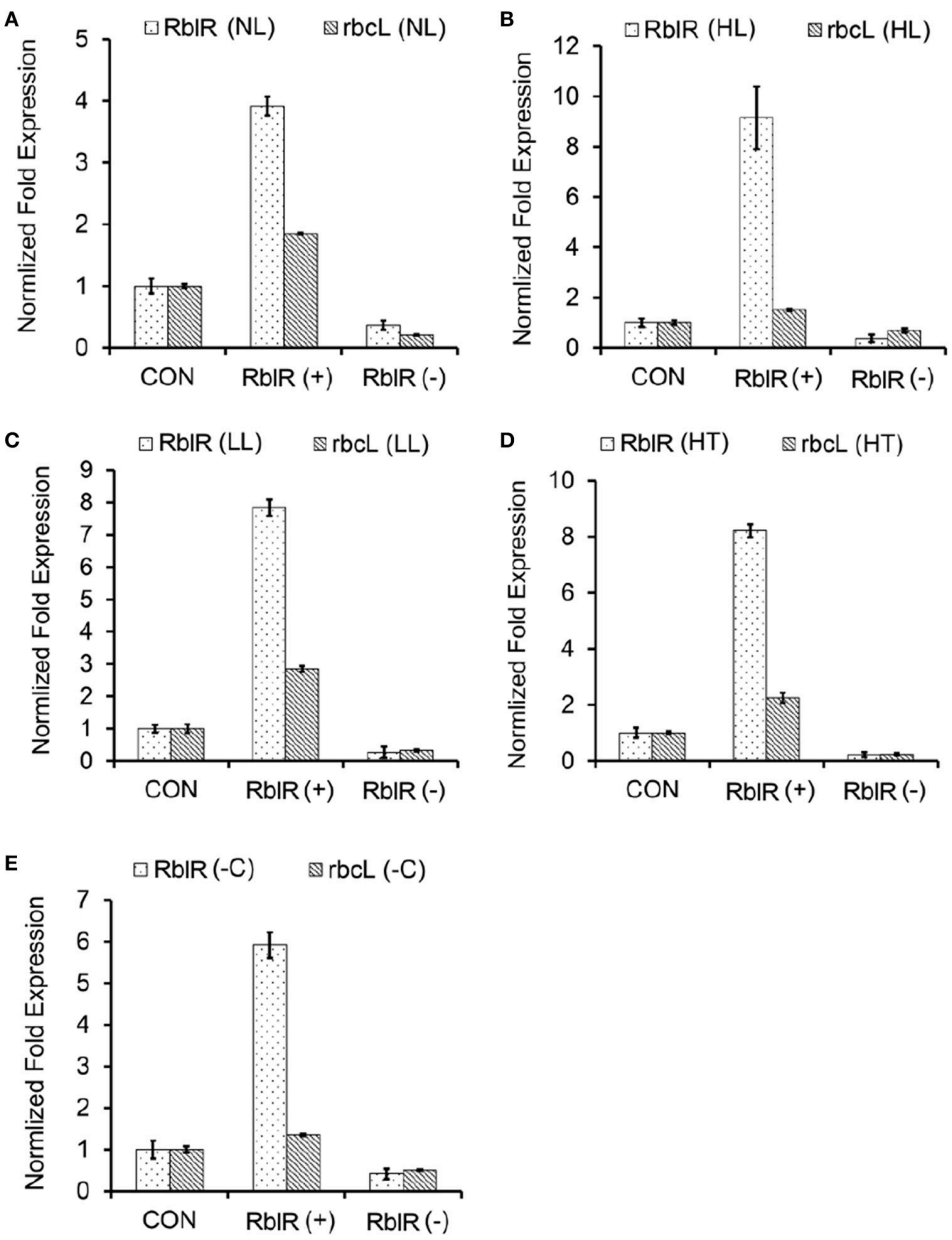
**Response of the expression of RblR and its target ***rbcL*** mRNA to various different conditions**. qRT-PCR analysis of RblR and *rbcL* mRNA levels in the control and RblR (+)/(−) mutant strains under NL (**A**, OD_730_ = 0.6), HL (**B**, 300 μE 12 h), LL (**C**, 2 μE 1 d), HT (**D**, 42°C 1 d), and -C (**E**, carbon-free BG11, 8 h) conditions. All data are shown as the means ±*SD* (*n* = 5).

**Figure 6:**
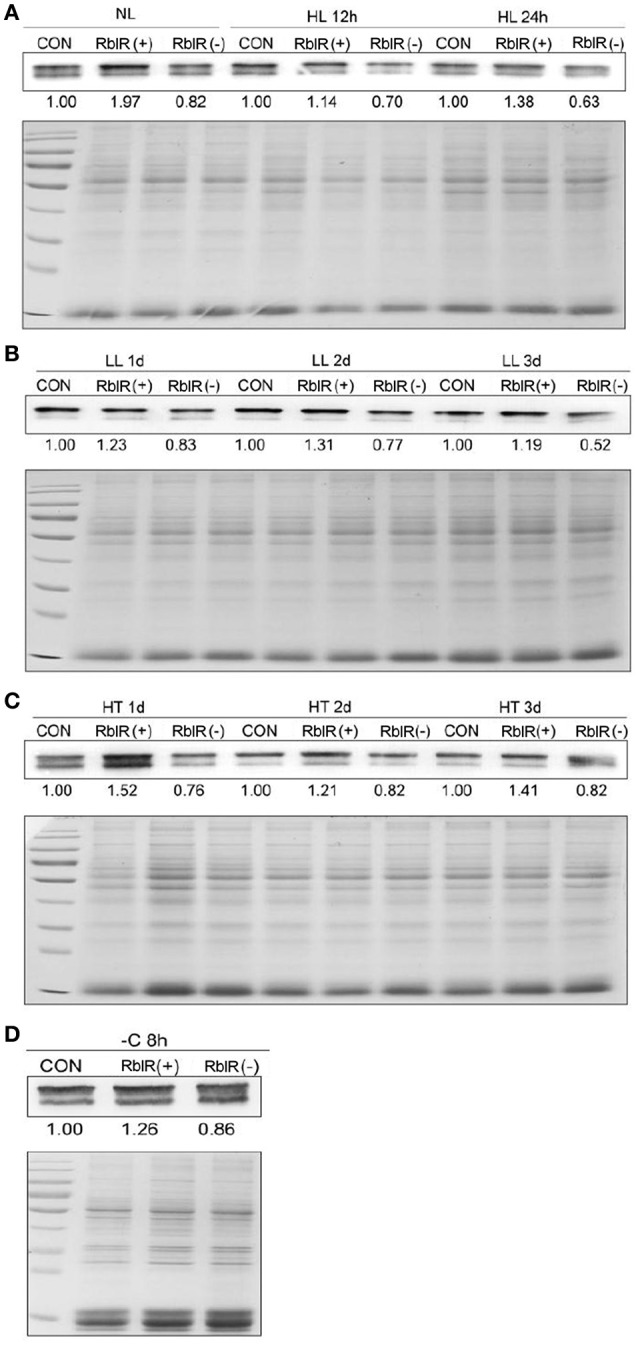
**Analysis of RbcL from RblR mutant strains**. Immunoblot analysis of RbcL protein in the control and RblR (+)/(−) mutant strains under NL (**A**, OD_730_ = 0.6), HL (**A**, 300 μE 12, 24 h), LL (**B**, 2 μE 1, 2, 3 days), HT (**C**, 42°C 1, 2, 3 days), and -C (**D**, carbon-free BG11, 8 h) conditions. The bottom panel is 20 μg total protein served as a loading control. Coomassie-stained sections of the polyacrylamide gel are shown. All data are shown as the means ±*SD* (*n* = 5).

RblR appears to have activating effects on *rbcL* expression, as revealed through qRT-PCR and immunoblot analyses of these mutant strains in the exponential growth phase (Figures 5A, 6A). To further investigate the effect of RblR on the expression of *rbcL* under different stress conditions related to photosynthesis (HL, LL, HT, −C), we analyzed the levels of RblR and *rbcL* mRNA and RbcL protein under various stress conditions (HL, LL, HT, −C).

As shown in Figure 5, the asRNA RblR levels were 3.92 (NL)-, 9.15 (HL)-, 7.84 (LL)-, 8.22 (HT)-, and 5.91 (−C)-fold in the RblR(+) strains but were only 0.36 (NL)-, 0.37 (HL)-, 0.26 (LL)-, 0.22 (HT)- and 0.41 (−C)-fold greater in the RblR(–) strains than in the control strains (Figure 5, white bars). The levels of *rbcL* mRNA in the RblR(+) strains under the above conditions increased by 1.85-, 1.51-, 2.85-, 2.25-, and 1.35-fold, whereas *rbcL* mRNA levels in the RblR(−) strains were 0.21-, 0.69-, 0.33-, 0.24-, and 0.51-fold lower than in the control strains (Figure 5, gray bars). Intriguingly, the levels of RbcL protein in the RblR(+) and RblR(−) strains were slightly different from the mRNA levels. The level of RbcL protein was 197% (NL), 138% (HL, 1 d), 131% (LL, 2 d), 152% (HT, 1 d), and 126% (−C, 8 h) that of control levels in the RblR(+) strains, whereas they were 82% (NL), 63% (HL, 1 d), 77% (LL, 2 d), 76% (HT, 1 d), and 86% (−C, 8 h) of control levels in RblR(−) strains (Figure 6). These results provide strong evidence that RblR, despite its low steady-state level, regulates photosynthesis by controlling *rbcL* expression in *Synechocystis*.

**Figure 7:**
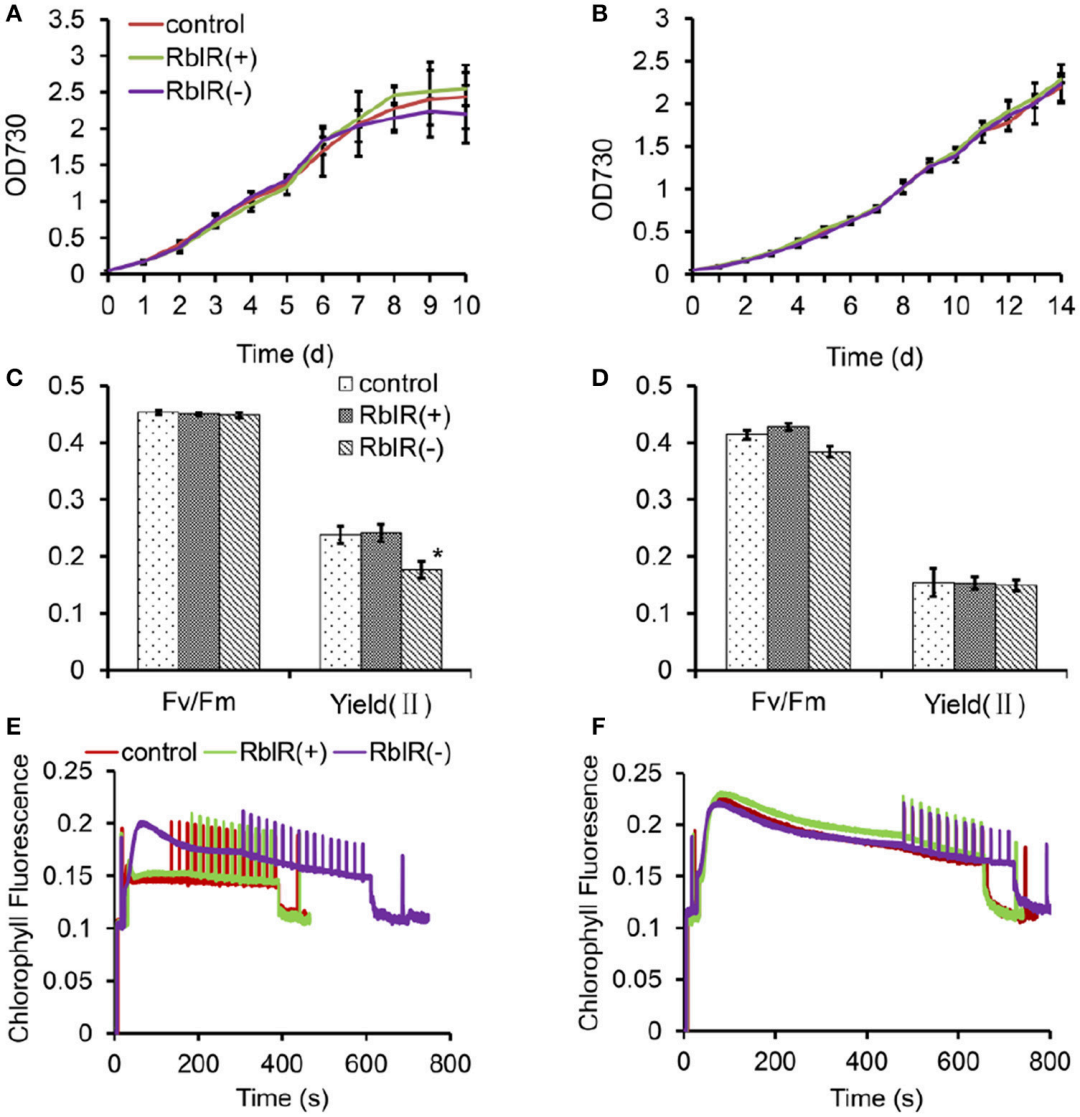
**The effect of RblR on the growth and photosynthesis of ***Synechocystis*** cells**. Fv/Fm, Yield (II), and Chl fluorescence induction traces were measured in NL **(A**,**C**,**E)** and −C **(B**,**D**,**F)** conditions, respectively. Measurements were performed in darkness at room temperature and saturation pulses were applied every 20 s. All data are shown as the means ±*SD* (*n* = 5).

To further determine the effects of RblR on growth and photosynthesis in *Synechocystis*, we analyzed the growth performances and photosynthetic activity in the three mutant strains under NL and -C conditions. Under NL conditions, although not significantly, the three mutant strains showed repeatable, variable growth rates with RblR(+) > control > RblR(−) in the exponential growth phase (Figure 7A). However, the growth phenotypes were lost under −C stress conditions, and the growth rates of all strains were much slower than those under NL conditions (Figure 7B).

Unsurprisingly, the maximum quantum yield of PS II in the dark, Fv/Fm, which represents the primary charge separation, was unaffected by the altered RblR levels (Figure 7C). While the effective quantum yield of PS II in the light, Yield(II), which represents the photochemical reactions and the following carbon fixation, was significantly reduced in the RblR(−) mutant strain (Figure 7C). Which could be further proved by the results that the chlorophyll fluorescence induction curve of the RblR(−) strain was different from that of both the control and the RblR(+) under NL conditions and took a lot longer (600 vs. 400 s) to reach its steady state (Figure 7E). However, both the Yield (II) and the fluorescence induction phenotype of both the control and the RblR(+) strains could be neutralized to the levels of the RblR(−) strain by carbon limitation stress (Figures 7D,F). These results indicate that suppression of RblR severely limits carbon fixation in the mutants compared to wild-type cell lines.

**Figure 8:**
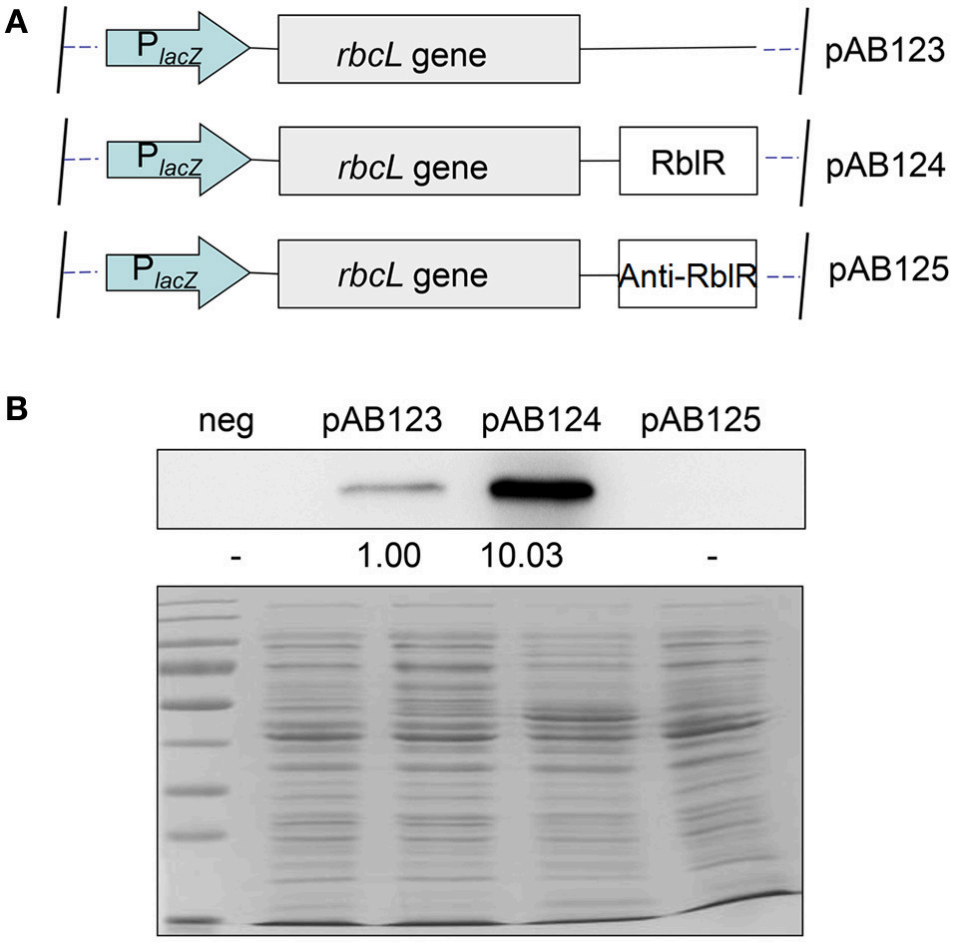
**Real-time simulation platform of RblR and ***rbcL*** in ***E. coli***. (A)** Both the *rbcL* gene and RblR under the control of a single *lacZ* promoter were built in a pMD18-T vector, pAB123: analogous to the control mutant; pAB123: analogous to the RblR(+) mutant; pAB125: analogous to the RblR(−) mutant. **(B)** Effect of RblR on the RbcL protein in *E. coli*. neg: negative control strain generated by the transformation of pMD18-T.

To further clarify the relationship between RblR and its target *rbcL* gene with a clear background, we setup a new expression platform to test the correlation between rbcL and RblR in *Escherichia coli* (Figure 8A). We placed both *rbcL* and RblR under the control of a single *lacZ* promoter in the pMD18-T vector (#6011, TAKARA, China). The strain pAB124, in which the level of the target protein RbcL was approximately ten-fold that in strain pAB123, exhibited extreme activation of RbcL expression by RblR (Figure 8B). Conversely, RbcL was almost undetectable in strain pAB125, as this strain was analogous to the negative control. These results suggest that the asRNA RblR has a positive effect on in the expression of the *rbcL* gene, and proved that the platform being a powerful tool in sRNA functional analysis.

**Figure 9:**
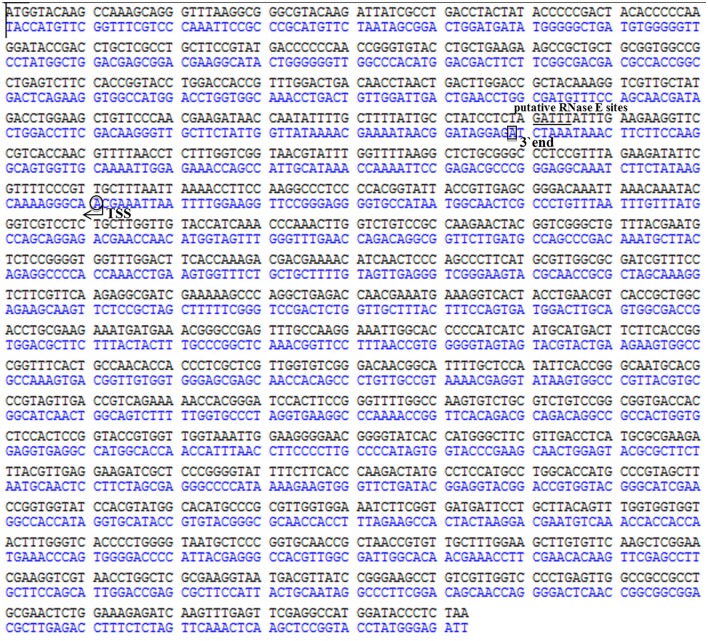
**Determination of the 5′ and 3′ ends of the asRNA RblR by RACE**. mRNA and asRNA are shown in the top and bottom DNA strands, respectively. Transcription start site (TSS) and the 3′ end of asRNA are indicated by an arrow in a circle and by a square, respectively. The putative RNase E site is indicated by a bar below the DNA strand. The start codon is indicated by a bar above the DNA strand.

In the current study, the consensus sequence GAUUU of RNase E sites was found at the N-terminal sequence of *rbcL* mRNA, which very possibly interacts with its asRNA RblR by complementary base pairing (Figure 9). We thus propose a mechanism in which the interaction of RblR and its complementary mRNA mask the RNase E cleavage sites and prevent RNase E-dependent degradation of the target mRNA.

## Discussion

The difference in sRNA contents under various conditions raises the question of their biological role in the organism. In the current study, transferring *Synechocystis* cultures to HL stress conditions influenced the expression levels of abundant sRNAs, suggesting their functional relevance to the HL stress response (Figure 1). HL stress has a significant impact on the photosynthetic apparatus (Demmig-Adams and Adams Iii, 1992). More than 160 differentially expressed genes were identified during acclimation from LL to HL in *Synechocystis* (Hihara et al., 2001). Mathematical modeling of sRNA-based gene regulation revealed a particular niche for regulatory RNA in allowing cells to transition quickly yet reliably between distinct states, which is consistent with the widespread appearance of sRNAs in stress regulatory networks (Mehta et al., 2008). In the current study, we sought to determine how sRNAs alter gene expression in *Synechocystis* under HL stress conditions. We found that sRNA expression is strongly affected by HL stress, resulting in distinct and characteristic changes in the expression of many sRNAs: one group of sRNAs (i.e., AS1, AS3, IGR1, and LR1) exhibited significant differential expression in both databases under NL and HL conditions, and another group of sRNAs (i.e., AS8, AS9, IGR3, and LR2) was detected in only one of the two databases (Supplementary Table 6, Figure 3). Collectively, these results suggest that sRNA-Seq studies can be used to analyze changes in the transcriptomes of bacteria subjected to different growth conditions. The comprehensive, unbiased profiles produced by sRNA-Seq will likely yield important insights into gene regulatory networks.

During carbon fixation, RuBisCO catalyzes the addition of an “activating” carbon dioxide molecule to a lysine at the active site (forming a carbamate) [9]. Since CO_2_ and O_2_ compete at the active site of RbcL, carbon fixation by RuBisCO can be enhanced by increasing the CO_2_ level in the carboxysome containing RuBisCO (Badger et al., 1998). This characteristic of the enzyme is the cause of photorespiration, a process in which healthy leaves subjected to HL fail to fix carbon when the O_2_/CO_2_ reaches a threshold at which oxygen is fixed instead of carbon. This phenomenon appears to be related to the fact that high temperatures reduce the concentration of CO_2_ dissolved in the moist leaf tissue (Brooks and Farquhar, 1985). The chloroplast *rbcL* gene, encoding the large subunit of RuBisCO, has binding sites for enzymatically active substrates and plays a central role in photosynthetic metabolism. Despite its relatively low abundance, we found that the asRNA RblR plays a substantial role in regulating *rbcL* expression and the photosynthetic network. As shown in Figure 7, both the Yield (II) and the chlorophyll fluorescence induction curve of the RblR(−) mutant strain were negatively affected, and those of both the control and the RblR(+) strains which were unaffected could be neutralized to the RblR(−) levels under limited-carbon conditions. These results indicate that the mutation in the RblR(−) mutant strain has a negative effect on carbon assimilation.

Our sRNA-Seq results suggest that a significant proportion of sRNAs are expressed from the reverse complementary strand of mRNA. Through complementary base pairing, asRNAs have multiple effects in bacteria, such as altering target mRNA stability, modulating translation, terminating transcription, and disrupting transcription (for reviews, see Georg and Hess, 2011). RNase, ribosomes, pH, inorganic carbon, and other unknown factors alter the regulatory effects of asRNAs on their targets, including negative or positive effects on gene expression (Kawano et al., 2007; Lee and Groisman, 2010; Opdyke et al., 2011; Stazic et al., 2011; Wen et al., 2011; Eisenhut et al., 2012). For instance, RNase plays a central role in RNA processing and decay and is involved in the degradation of most mRNAs. The sequence of the N-terminal endoribonucleolytic domain of RNase E is evolutionarily conserved in *Synechocystis* sp. and other bacteria (Kaberdin et al., 1998). These findings and an analysis of all known putative RNase E sites suggest the presence of the consensus sequence RAUUW (R = A or G; W = A or U) at the cleavage site (Ehretsmann et al., 1992). Two cis-encoded asRNAs, named PsbA2R and PsbA3R, are located in the 5′ untranslated region (5′UTR) of *psbA2* and *psbA3* genes in *Synechocystis* sp. PCC 6803, which encode the D1 protein of photosystem II in the thylakoid membrane (Sakurai et al., 2012). PsbA2R has a capacity to protect the AU box by duplex formation with the *psbA2* mRNA, which is cleaved by RNase E at AU box and RBS both located in the 5′ UTR of the mRNA. In this study, the consensus sequence GAUUU of RNase E sites was found at the N-terminal sequence of *rbcL* mRNA, which very possibly interacts with its asRNA RblR by complementary base pairing (Figure 9). We thus propose a mechanism in which the interaction of RblR and its complementary mRNA mask the RNase E cleavage sites and prevent RNase E-dependent degradation of the target mRNA. This idea is based on the current results and a previous hypothesis describing the interplay between asRNA RblR, the target *rbcL* mRNA, and RNase E (Stazic et al., 2011). The results obtained in this study provide new insights into the interaction between sRNAs and their targets.

In summary, asRNA of *rbcL* gene, RblR is 113 nt in length and completely complementary to its target gene *rbcL*, which encodes the large chain of RuBisCO, the enzyme that catalyzes carbon fixation. This asRNA is found in low abundance in the cell, yet it is shown here to act as an important negative regulator of RbcL protein that maintains the functionality of RuBisCO in *Synechocystis* sp. PCC 6803. A mechanism was proposed in which the interaction of RblR and its complementary mRNA mask the RNase E cleavage sites and prevent RNase E-dependent degradation of the target mRNA. The results obtained in this study add a new layer of complexity to the mechanisms that contribute to the regulation of *rbcL* gene expression.

## Author contributions

QW contributed to the conception or design of the study, interpretation of the data and writing of the manuscript; JH contributed to the acquisition, analysis, interpretation of the data; and writing of the manuscript: TL, WX, JZ, HC, and CH contributed to the acquisition, analysis, or interpretation of the data.

## Funding

This work was supported jointly by the National Program on Key Basic Research Project (2012CB224803), the National Natural Science Foundation of China (31270094) and the State Key Laboratory of Freshwater Ecology and Biotechnology (Y11901-1-F01).

### Conflict of interest statement

The authors declare that the research was conducted in the absence of any commercial or financial relationships that could be construed as a potential conflict of interest.

## Supplementary material

Supplementary Table 1 **Oligonucleotides used for northern blot analysis**.

Supplementary Table 2 **Oligonucleotides used for RACE analysis**.

Supplementary Table 3 **Primers used for qRT-PCR of eight antisense RNAs and their targets**.

Supplementary Table 4 **Primers used for RblR analysis**.

Supplementary Table 5 **Differential expression libraries from the NL and HL libraries depending on three constraints: overlap ≥10%, ratio ≥2, reads ≥10 in the NL/HL condition**. Differential expression libraries include three types of sRNA libraries. (1). Up/down-regulated sRNAs in the NL library compared with the HL library; (2). Unique sRNAs in the NL library; (3). Unique sRNAs in the HL library.

Supplementary Table 6 **Selected novel and established sRNAs**. The coordinates and length of each candidate represent the most abundant sequence based on dRNA-Seq data and RNA blot analysis; (+) plus strand; (−) minus strand. Transcription start sites (TSSs) and the 3′ ends of sRNAs, as determined by 5′ RACE and 3′ RACE. The list has been sorted according to the type of sRNA candidates and their location in the Synechocystis genome; –, none detected.

Supplementary Figure S1 **Type distribution of sRNA candidates under NL and HL conditions (reads ≥10)**. Antisense sRNA (asRNA), intergenic region-sRNA (IGR), and 5′ leader region-sRNA (5′ LR).

Supplementary Figure S2 **Level of eight identified asRNAs and their target mRNAs under NL and HL conditions**. qRT-PCR analysis of asRNAs and their target mRNAs levels in *Synechocystis* under NL and HL conditions. AS1-*sll0247* mRNA **(a)**, AS2-*sll1507* mRNA **(b)**, AS3-*slr0534* mRNA **(c)**, AS4-*slr009* mRNA **(d)**, AS5-*slr2017* mRNA **(e)**, AS6-*slr1324* mRNA **(f)**, AS8-*ssl5070* mRNA **(g)**, and AS9-*slr0869* mRNA **(h)**.

Supplementary Figure S3 **Overexpression and suppression of RblR. (a)** The sense and antisense fragments of RblR were fused to the *rnpB* and *rbcL* promotor, yielding overexpressor RblR(+) and suppressor RblR(−), respectively. The control strain contains only the *kanamycin* resistance cassette. **(b)** Schematic diagram of RblR(+)/(−) mutant construction. **(c)** Segregation analysis of transformants. Disruption of the *slr0168* region was evaluated by PCR. WT, wild type.
