# Supplementary material for: Small Antisense RNA RblR Positively Regulates RuBisCo in Synechocystis sp. PCC 6803
Source: Front Microbiol. 2017 Feb 14;8:231. doi: 10.3389/fmicb.2017.00231 (PMC5306279; doi:10.3389/fmicb.2017.00231)
Supplement: Supplementary Table 1 — Oligonucleotides used for northern blot analysis. [file Table1.DOCX]

**Supplementary Table 1.** Oligonucleotides used for northern blot analysis

| **DNA oligonucleotide (5’-3’ sequence)** | |
| --- | --- |
| Peak6304**6** | GGCAGGCAAAGCCATGTATTGGGGG |
| Peak1812 | CCCTTCTAAGCGGGACAATAAA |
| Peak13495 | AGTCCGTCCTTGGCAGCAGA |
| Peak10444 | CGTATTTGGTTTTAAGGCTCTGC |
| Peak3273 | TTGGAGTTACGGGAAACCTTAC |
| Peak7093 | CATGTGCAAAGGTTGGGGG |
| Peak6620 | TTTACACTCTGTTAGCTGACCAGGA |
| Peak298 | CAGTCTCTCGCTCAATGTTCAAATC |
| Peak5796 | CAATGCGTTGTTGGAGCGG |
| Peak10276 | GGGCCTTGCTGACCATACTAAAT |
| Peak169 | CGGGAACCCTTTGTTTAGTGGA |
| Peak76 | GCGACTAGAACAGCAAGCGG |
| Peak11154 | GGGCCTTGCTGACCATACTAAAT |
| Peak7556 | GAGTTCACGCCTACCACAAGCATC |
