# Supplementary material for: Small Antisense RNA RblR Positively Regulates RuBisCo in Synechocystis sp. PCC 6803
Source: Front Microbiol. 2017 Feb 14;8:231. doi: 10.3389/fmicb.2017.00231 (PMC5306279; doi:10.3389/fmicb.2017.00231)
Supplement: Supplementary Table 2 — Oligonucleotides used for RACE analysis. [file Table2.DOCX]

**Supplementary Table 2.** Oligonucleotides used for RACE analysis

| **DNA oligonucleotide (5’-3’ sequence)** | | |
| --- | --- | --- |
| 3’ linker | phosphorylated-AAGATGAATGCAACACTTCTGTACGACTAGAGCAC-NH_2_ | RACE |
| 3’ RTrevlinker | GTGCTCTAGTCGTACAGAAGTGTTGCATTCATC | RACE |
| 3’ PCRrevlinker | GTGCTCTAGTCGTACAGAAGTGTTGCATTCATC | RACE |
| Peak6304PCRrev | CCCCCAGCAACTAGGGATAA | 5’ RACE |
| Peak6304PCR2rev | GGGCCGGAGCTCTACTGTTG | 5’ RACE |
| Peak6304PCR3rev | GCAGGCAAAGCCATGTATTG | 5’ RACE |
| Peak10444PCRrev | ATTTATTTGAAGAAGGTTCCGTC | 5’ RACE |
| Peak10444PCR2rev | ACGTTTTAACCTCTTTGGTCGG | 5’ RACE |
| Peak10444PCR3rev | TAACGTATTTGGTTTTAAGGCTCT | 5’ RACE |
| Peak3273PCRrev | TAATAATTACACTGCCGGCGG | 5’ RACE |
| Peak3273PCR2rev | ACGGGGCTATCGCCTCAA | 5’ RACE |
| Peak3273PCR3rev | GCAAGCAACTATTGATGGGGT | 5’ RACE |
| Peak7093PCRrev | ACAGGGGGAAAAGTTTGACATTA | 5’ RACE |
| Peak7093PCR2rev | TAACCCTACCGGACAGCCAAG | 5’ RACE |
| Peak7093PCR3rev | AACTGCAAAGCCATCCTCAA | 5’ RACE |
| Peak6620PCRrev | CACTCTGTTAGCTGACCAGGACC | 5’ RACE |
| Peak6620PCR2rev | ACCCAGAGGTGAAGGACAATG | 5’ RACE |
| Peak6620PCR3rev | GACAATGACGAAAAACGTCAAGA | 5’ RACE |
| Peak11154PCRrev | GGCAAGAAAAAGCCCCTGGG | 5’ RACE |
| Peak11154PCR2rev | CCTGGGCGTCTTTCCTTGTTTC | 5’ RACE |
| Peak11154PCR3rev | CTTTCCTTGTTTCGACTCCGG | 5’ RACE |
| Peak7556PCRrev | CAGAAAAGCCGGAAGCCC | 5’ RACE |
| Peak7556PCR2rev | GAGTTCACGCCTACCACAAGCATC | 5’ RACE |
| Peak6304PCRfw | AGGATCTAATGTTGGCTGACTGAC | 3’ RACE |
| Peak6304PCR2fw | CCGTTTGGGTGGTGGCGTC | 3’ RACE |
| Peak10444PCRfw | AATTAAAGCAACGGGAAAACGAA | 3’ RACE |
| Peak10444PCR2fw | ACGGAGGGCCCGCAGAGC | 3’ RACE |
| Peak3273PCRfw | GGAAGAAGTCTAGTGCAATCGGA | 3’ RACE |
| Peak3273PCR2fw | ATTGAGGCGATAGCCCCGTTC | 3’ RACE |
| Peak7093PCRfw | GCGAACGTAGGAGAATATCCAAG | 3’ RACE |
| Peak7093PCR2fw | GGATGGCTTTGCAGTTTCG | 3’ RACE |
| Peak6620PCRfw | TTTCGTCATTGTCCTTCACCTC | 3’ RACE |
| Peak6620PCR2fw | CTCTGGGTCCTGGTCAGCTAAC | 3’ RACE |
| Peak11154PCRfw | GGTGTGTGAGGAGTAAGTTGAAGC | 3’ RACE |
| Peak11154PCR2fw | GAAGCCCCCGGAGTCGAAAC | 3’ RACE |
| Peak7556PCRfw | CGGACTCATGCGATTACAAC | 3’ RACE |
| Peak7556PCR2fw | CAAATCTTGTCAGGACCGGA | 3’ RACE |

The 3’ RACE analysis was performed prior to 5’ RACE. All PCR primers were designed according to the results of 3’ RACE.
