# Supplementary material for: Small Antisense RNA RblR Positively Regulates RuBisCo in Synechocystis sp. PCC 6803
Source: Front Microbiol. 2017 Feb 14;8:231. doi: 10.3389/fmicb.2017.00231 (PMC5306279; doi:10.3389/fmicb.2017.00231)
Supplement: Supplementary Table 3 — Primers used for qRT-PCR of eight antisense RNAs and their targets. [file Table3.DOCX]

**Supplementary Table 3.** Primers used for qRT-PCR of eight antisense RNAs and their targets

| **qRT-PCR primer (5’-3’ sequence)** | |
| --- | --- |
| Peak6304-F | AGGATCTAATGTTGGCTGACTGAC |
| Peak6304-R | GGTCATCATCTCCTGTTCCTGG |
| Peak1812-F | GCTTCTTTATTGTCCCGCTTAG |
| Peak1812-R | CGTTTCAGGCAGATCCAGTTAC |
| Peak13495-F | CAACGGCAACATTGGCTTTA |
| Peak13495-R | CCGAATTTATCTAGTCCGTCCTT |
| Peak298-F | GAAATGCCACCGTAGGACTTG |
| Peak298-R | CTCTCGCTCAATGTTCAAATCA |
| Peak5796-F | CGTGGCGTGACCTTCTGC |
| Peak5796-R | ATTGTTTTTGCCGGGGCT |
| Peak10444-F | CGGGAAAACGAATATCTTCTAAAC |
| Peak10444-R | TAGATTTATTTGAAGAAGGTTCCG |
| Peak3273-F | GAAGTCTAGTGCAATCGGAAGG |
| Peak3273-R | TTGGAGTTACGGGAAACCTTAC |
| Peak7093-F | ACGTAGGAGAATATCCAAGCTCAC |
| Peak7093-R | CATGTGCAAAGGTTGGGGG |
| Peak5796-F | CGTGGCGTGACCTTCTGC |
| Peak5796-R | ATTGTTTTTGCCGGGGCT |
| sll0247-F | TTTGCCGATACGGTGGAACT |
| sll0247 -R | AAACCCAAAGCCCGCAGT |
| sll1507-F | CCCTTCTAAGCGGGACAATA |
| sll1507-R | GGAGTTCTGGGGTGGGATAA |
| slr0534-F | CGGTAAACGCTGGTTCTGG |
| slr0534-R | CTTGGTGTTGTTCCTGCTCG |
| ssl5070-F | TTGCTCACTCAATTTGACCTT |
| ssl5070-R | TGGATCCGCAGGGATAATAAT |
| slr0869-F | CTATTACGACGGGCGGAAG |
| slr0869-R | CCCTGGGAAGTTTGCTGGA |
| slr0009-F | GGTCGTCCTCTGCTTGGTTGTA |
| slr0009-R | TGAAGGGCTGGGAGTTGATGT |
| slr2017-F | CAGATTGAGGGTTTGTGAAGA |
| slr2017-R | GTTGCCGCCATTGCTCTC |
| slr1324-F | TAAACGCCAGGAAAGGGA |
| slr1324-R | ATTATGTTGTTGCCCTTTACTCA |
| rnpb-F | CGGTTGGAAGCAAGGTCG |
| rnpb-R | AAGAGAGTTAGTCGTAAGCCG |
