# Supplementary material for: Small Antisense RNA RblR Positively Regulates RuBisCo in Synechocystis sp. PCC 6803
Source: Front Microbiol. 2017 Feb 14;8:231. doi: 10.3389/fmicb.2017.00231 (PMC5306279; doi:10.3389/fmicb.2017.00231)
Supplement: Supplementary Table 4 — Primers used for RblR analysis. [file Table4.DOCX]

**Supplementary Table 4.** Primers used for RblR analysis

| **PCR primer (5’-3’ sequence)** | |
| --- | --- |
| slr0168-F | ACCTCTCCACGCTGAATTAGA |
| slr0168-R | TAATACCCACCGCACTGACC |
| 5’rnpB | AATGCGGTCCAATACCTCC |
| 3**’**rnpB/kana | GTTACCCAACTGATATCTCTTTTTCTAGTGTGCCATTG |
| 5**’**kana/rnpB | CTAGAAAAAGAGATATCAGTTGGGTAACGCCAGGG |
| 3**’**kana | CACTTTATGCTTCCGGCTCG |
| RbclR(+)-F | CGGGAAAACGAATATCTTCTAAAC |
| RbclR(+)-R | TAGATTTATTTGAAGAAGGTTCCG |
| RbclR(-)-F | TTTATTTGAAGAAGGTTCCGTC |
| RbclR(-)/oop ter-R | ggaataaaaaacgcccggcggcaaccgagcgttAACGGGAAAACGAATATCTTC |
| Prbcl-F | C GAGCTC CCGATGAAGTGGTGGAGCA |
| Prbcl-R | GC TCTAGA GGTCAGTCCTCCATAAACATTG |
| 0168-F | CCCTGAAGTTAGCCAGTTTAATTG |
| 0168-R | GTCACTGAAGCGGTCTAACTTAGC |
| rbcL-F | agatctagatgactaaggagatatacat ATGGTACAAGCCAAAGCAGG |
| rbcL-R | TTAGAGGGTATCCATGGCCTC |
