# Supplementary material for: Small Antisense RNA RblR Positively Regulates RuBisCo in Synechocystis sp. PCC 6803
Source: Front Microbiol. 2017 Feb 14;8:231. doi: 10.3389/fmicb.2017.00231 (PMC5306279; doi:10.3389/fmicb.2017.00231)
Supplement: Supplementary Table 5 — Differential expression libraries from the NL and HL libraries depending on three constraints: overlap ≥10%, ratio ≥2, reads ≥10 in the NL/HL condition. Differential expression libraries include three types of sRNA libraries. (1). Up/down-regulated sRNAs in the NL library compared with the HL library; (2). Unique sRNAs in the NL library; (3). Unique sRNAs in the HL library. [file Table5.DOCX]

**Supplementary Table 5.** Differential expression libraries from the NL and HL libraries depending on three constraints: overlap ≥10%, ratio ≥2, reads ≥10 in the NL/HL condition. Differential expression libraries include three types of sRNA libraries. 1). Up/down-regulated sRNAs in the NL library compared with the HL library; 2). Unique sRNAs in the NL library; 3). Unique sRNAs in the HL library.

| **sRNA types** | **asRNA** | **IGR** | **5’ LR** | **Total** |
| --- | --- | --- | --- | --- |
| Up-regulated sRNAs | 764 | 63 | 26 | 853 |
| Down-regulated sRNAs | 2362 | 136 | 50 | 2548 |
| sRNAs in NL conditions | 1010 | 199 | 110 | 1319 |
| sRNAs in HL conditions | 541 | 96 | 33 | 670 |
