# Supplementary material for: Small Antisense RNA RblR Positively Regulates RuBisCo in Synechocystis sp. PCC 6803
Source: Front Microbiol. 2017 Feb 14;8:231. doi: 10.3389/fmicb.2017.00231 (PMC5306279; doi:10.3389/fmicb.2017.00231)
Supplement: Supplementary Table 6 — Selected novel and established sRNAs. The coordinates and length of each candidate represent the most abundant sequence based on dRNA-Seq data and RNA blot analysis; (+) plus strand; (−) minus strand. Transcription start sites (TSSs) and the 3′ ends of sRNAs, as determined by 5′ RACE and 3′ RACE. The list has been sorted according to the type of sRNA candidates and their location in the Synechocystis genome; –, none detected. [file Table6.DOCX]

| **Name** | **Coordinates** | **NB** | **TSS** | **3’ end** | **Annotation** | **Type** | **Location** |
| --- | --- | --- | --- | --- | --- | --- | --- |
| AS1 | 1518020-1518212 (+) | 177 | 1518032 | 1518209 | as_sll0247/isrR | asRNA | Chromosome |
| AS2 | 469091-469311 (+) | 220 | - | - | as_sll1507 | asRNA | Chromosome |
| AS3 | 3194103-3194339 (-) | 240 | - | - | as_slr0534 | asRNA | Chromosome |
| AS4 | 2478727-2478824 (-) | 113 | c2478718 | c2478830 | as_slr0009 | asRNA | Chromosome |
| AS5 | 758301-758556 (-) | 210 | c758305 | c758514 | as_slr2017 | asRNA | Chromosome |
| AS6 | 1653552-1653837 (-) | 252 | c1653556 | c1653807 | as_slr1324 | asRNA | Chromosome |
| AS7 | 1603665-1603753 (+) | 122/83 | 1603634 | 1603755/1603716 | as_sll1414 | asRNA | Chromosome |
| AS8 | 71192-71427 (+) | 230 | - | - | as_ssl5070 | asRNA | pSYSM |
| AS9 | 1367239-1367410 (-) | 180 | - | - | as_slr0869 | asRNA | Chromosome |
| IGR1 | 2530895-2530962 (+) | 60 | - | - | slr0228>>sll0195 | IGR | Chromosome |
| IGR2 | 39449-39678 (+) | 230 | - | - | sll7044>>ssl7045 | IGR | pSYSA |
| IGR3 | 15392-15481 (+) | 90 | 15410 | 15482 | sll8018>>sll8019 | IGR | pSYSG |
| LR1 | 2730521-2730591 (+) | 70 | 2730523 | 2730626 | Yfr2b | 5’ LR | Chromosome |
| LR2 | 1832226-1832339 (+) | 100 | 1832234 | 1832327 | SRP RNA ffs | 5’ LR | Chromosome |

**Supplementary Table 6.** Selected novel and established sRNAs. The coordinates and length of each candidate represent the most abundant sequence based on dRNA-Seq data and RNA blot analysis; (+) plus strand; (-) minus strand. Transcription start sites (TSSs) and the 3’ ends of sRNAs, as determined by 5’ RACE and 3’ RACE. The list has been sorted according to the type of sRNA candidates and their location in the Synechocystis genome. -, none detected.
