# Supplementary figures and images for: Small Antisense RNA RblR Positively Regulates RuBisCo in Synechocystis sp. PCC 6803
Source: Front Microbiol. 2017 Feb 14;8:231. doi: 10.3389/fmicb.2017.00231 (PMC5306279; doi:10.3389/fmicb.2017.00231)

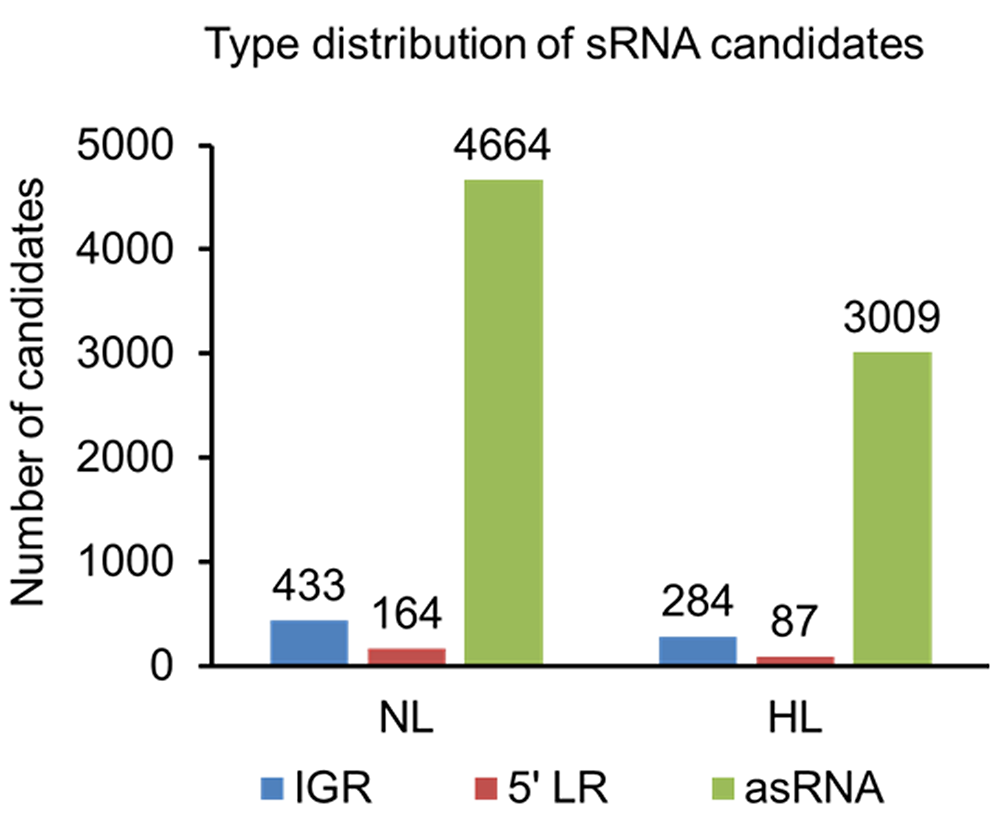

Supplement: Supplementary Figure S1 — Type distribution of sRNA candidates under NL and HL conditions (reads ≥10). Antisense sRNA (asRNA), intergenic region-sRNA (IGR), and 5′ leader region-sRNA (5′ LR). [file Image1.TIF]

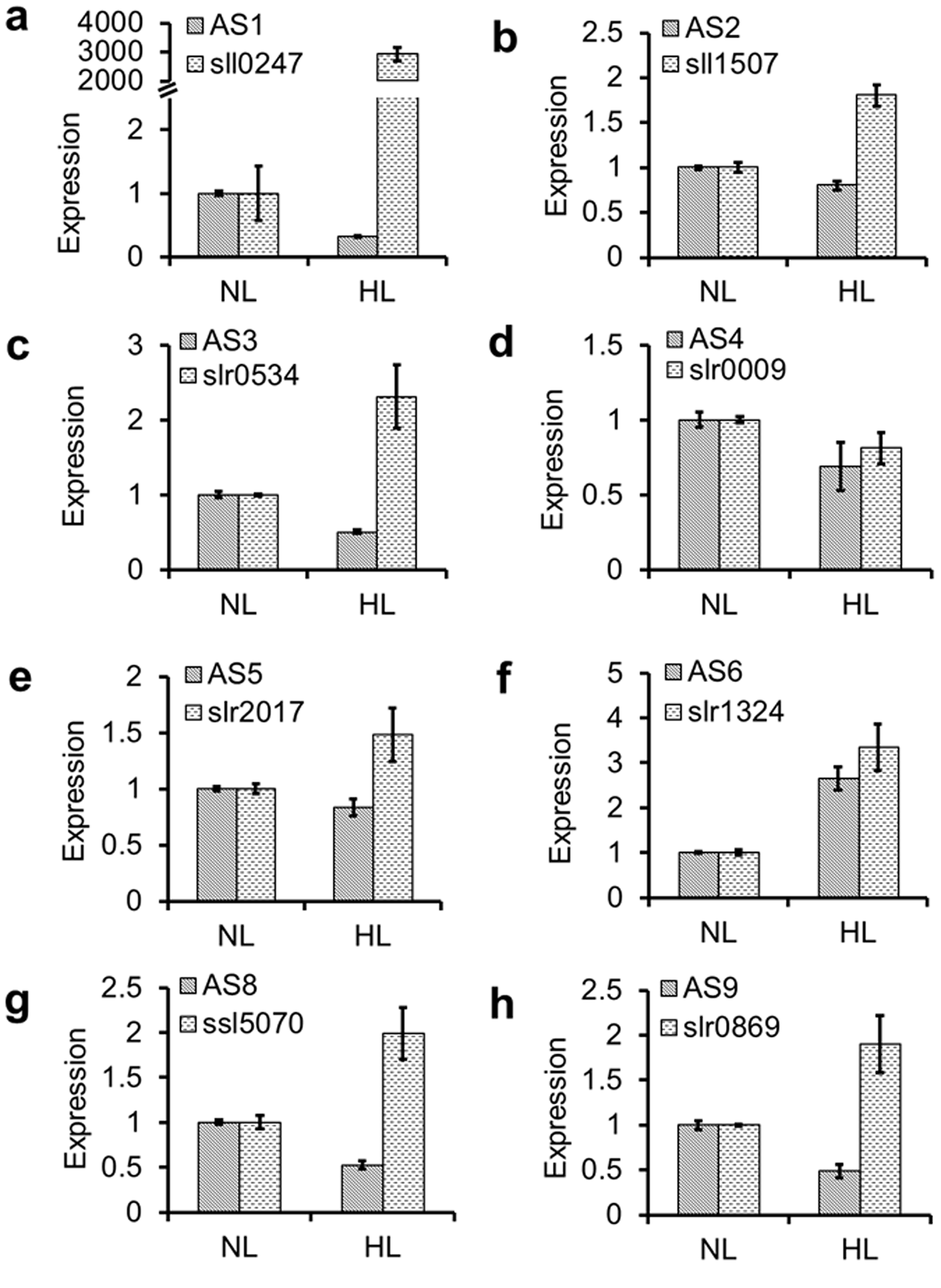

Supplement: Supplementary Figure S2 — Level of eight identified asRNAs and their target mRNAs under NL and HL conditions. qRT-PCR analysis of asRNAs and their target mRNAs levels in Synechocystis under NL and HL conditions. AS1-sll0247 mRNA (a), AS2-sll1507 mRNA (b), AS3-slr0534 mRNA (c), AS4-slr009 mRNA (d), AS5-slr2017 mRNA (e), AS6-slr1324 mRNA (f), AS8-ssl5070 mRNA (g), and AS9-slr0869 mRNA (h). [file Image2.TIF]

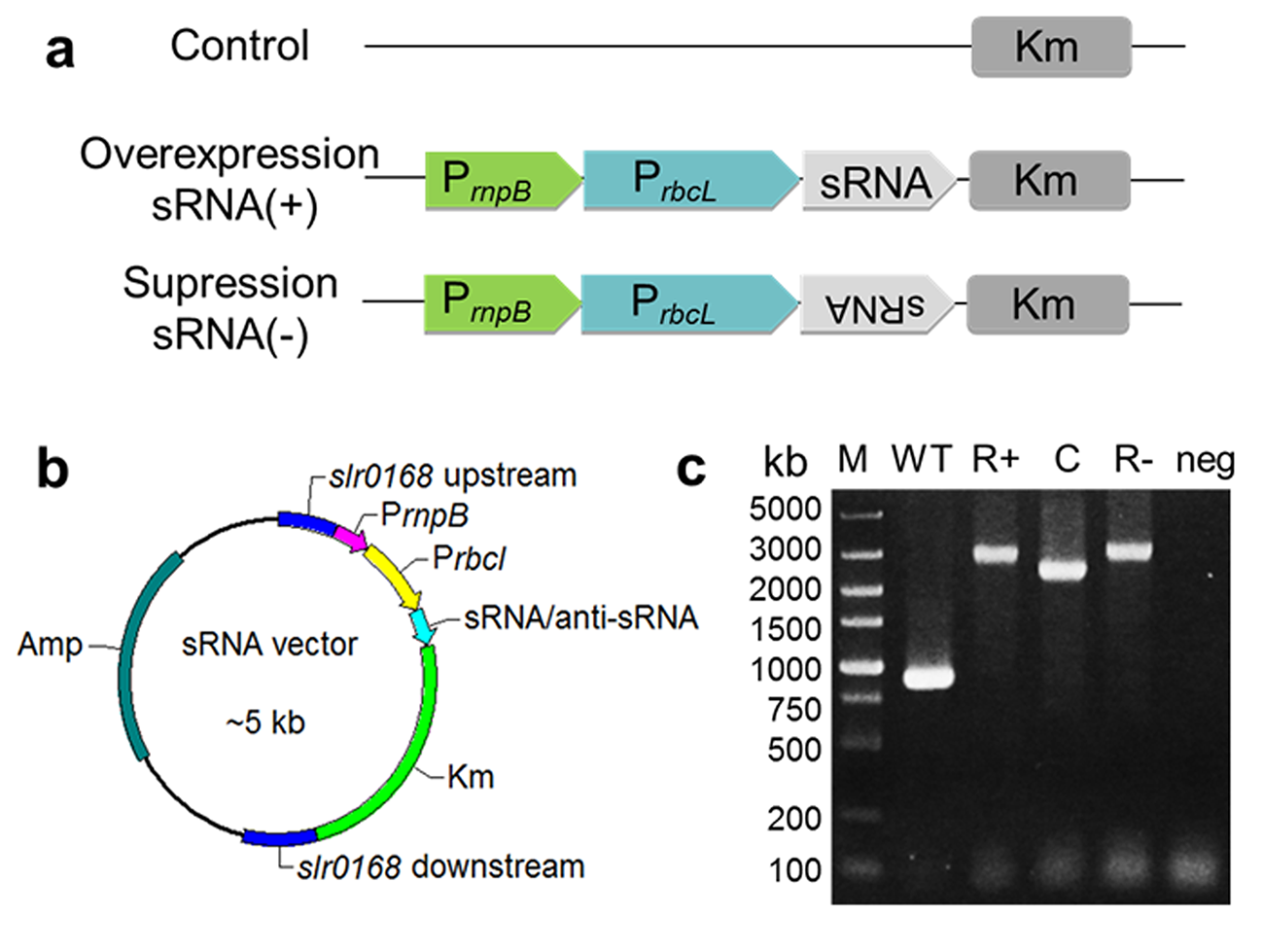

Supplement: Supplementary Figure S3 — Overexpression and suppression of RblR. (a) The sense and antisense fragments of RblR were fused to the rnpB and rbcL promotor, yielding overexpressor RblR(+) and suppressor RblR(−), respectively. The control strain contains only the kanamycin resistance cassette. (b) Schematic diagram of RblR(+)/(−) mutant construction. (c) Segregation analysis of transformants. Disruption of the slr0168 region was evaluated by PCR. WT, wild type. [file Image3.TIF]
